# 4,4′-Dichloro-2,2′-[2,2-dimethyl­propane-1,3-diylbis(nitrilo­methyl­idyne)]diphenol

**DOI:** 10.1107/S1600536808038014

**Published:** 2008-12-10

**Authors:** Chin Sing Yeap, Hadi Kargar, Reza Kia, Hoong-Kun Fun

**Affiliations:** a X-ray Crystallography Unit, School of Physics, Universiti Sains Malaysia, 11800 USM, Penang, Malaysia; b Department of Chemistry, School of Science, Payame Noor University (PNU), Ardakan, Yazd, Iran

## Abstract

The crystal of the title Schiff base compound, C_19_H_20_Cl_2_N_2_O_2_, contains of two crystallographically independent mol­ecules with similar conformations. In each mol­ecule, two intramolecular O—H⋯N bonds generate *S*(6) motifs. The N atoms are also in close proximity to two H atoms of the dimethyl­propane groups, with H⋯N distances between 2.59 and 2.62 Å. The imine group is coplanar with the benzene ring. The dihedral angles between the benzene rings in the two independent mol­ecules are 58.20 (12) and 47.95 (12)°. The structure displays short inter­molecular Cl⋯Cl [3.3869 (11) Å] and Cl⋯O [3.175 (2)–3.204 (2) Å] inter­actions. The crystal structure is further stabilized by weak inter­molecular C—H⋯O, C—H⋯π and π–π [centroid–centroid distances 3.6416 (13)–3.8705 (14) Å] inter­actions.

## Related literature

For the values of bond lengths, see: Allen *et al.* (1987 ▶). For hydrogen-bond motifs, see: Bernstein *et al.* (1995 ▶). For information on Schiff base ligands and complexes and their applications, see: Calligaris & Randaccio (1987 ▶); Casellato & Vigato (1977 ▶). For similar structures, see: Bomfim *et al.* (2005 ▶); Fun *et al.* (2008 ▶); Glidewell *et al.* (2005 ▶, 2006 ▶); Li *et al.* (2005 ▶); Sun *et al.* (2004 ▶).
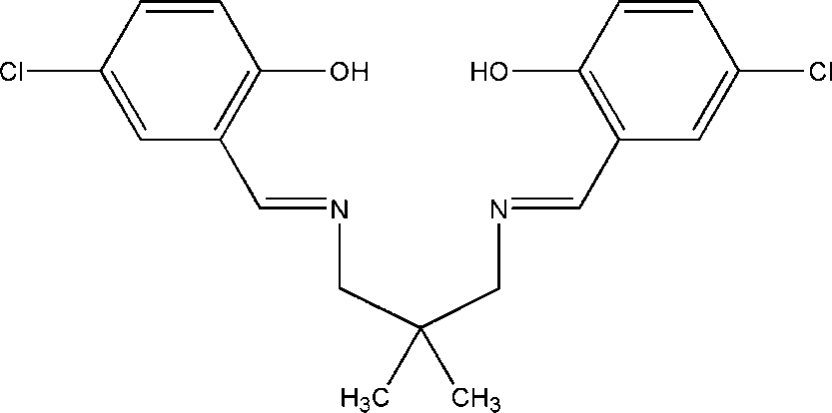


## Experimental

### 

#### Crystal data

C_19_H_20_Cl_2_N_2_O_2_
*M* *_r_* = 379.27
Monoclinic, 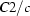

*a* = 31.6843 (8) Å
*b* = 6.2236 (2) Å
*c* = 37.9015 (10) Å
β = 99.779 (1)°
*V* = 7365.2 (4) Å^3^
*Z* = 16
Mo *K*α radiation
μ = 0.37 mm^−1^
*T* = 100.0 (1) K
0.35 × 0.06 × 0.04 mm

#### Data collection

Bruker SMART APEXII CCD area-detector diffractometer
Absorption correction: multi-scan (**SADABS**; Bruker, 2005 ▶) *T*_min_ = 0.882, *T*_max_ = 0.986
38685 measured reflections
8427 independent reflections
5995 reflections with *I* > 2σ(*I*)
*R*_int_ = 0.071

#### Refinement

*R*[*F* ^2^ > 2σ(*F* ^2^)] = 0.061
*wR*(*F* ^2^) = 0.116
*S* = 1.12
8426 reflections
467 parameters
H atoms treated by a mixture of independent and constrained refinement
Δρ_max_ = 0.36 e Å^−3^
Δρ_min_ = −0.28 e Å^−3^

### 

Data collection: *APEX2* (Bruker, 2005 ▶); cell refinement: *SAINT* (Bruker, 2005 ▶); data reduction: *SAINT*; program(s) used to solve structure: *SHELXS97* (Sheldrick, 2008 ▶); program(s) used to refine structure: *SHELXL97* (Sheldrick, 2008 ▶); molecular graphics: *SHELXTL* (Sheldrick, 2008 ▶); software used to prepare material for publication: *SHELXTL* and *PLATON* (Spek, 2003 ▶).

## Supplementary Material

Crystal structure: contains datablocks global, I. DOI: 10.1107/S1600536808038014/is2362sup1.cif

Structure factors: contains datablocks I. DOI: 10.1107/S1600536808038014/is2362Isup2.hkl

Additional supplementary materials:  crystallographic information; 3D view; checkCIF report

## Figures and Tables

**Table 1:** Hydrogen-bond geometry (Å, °)

*D*—H⋯*A*	*D*—H	H⋯*A*	*D*⋯*A*	*D*—H⋯*A*
O1*A*—H1*OA*⋯N1*A*	0.92 (4)	1.76 (4)	2.594 (3)	150 (4)
O2*A*—H2*OA*⋯N2*A*	0.86 (4)	1.82 (4)	2.591 (3)	148 (3)
O1*B*—H1*OB*⋯N1*B*	0.84 (4)	1.80 (4)	2.579 (3)	153 (3)
O2*B*—H2*OB*⋯N2*B*	0.82 (4)	1.85 (4)	2.595 (3)	151 (4)
C16*A*—H16*A*⋯O2*A*^i^	0.95	2.54	3.291 (3)	136
C18*A*—H18*C*⋯*Cg*1	0.98	2.73	3.634 (3)	153

Symmetry code: (i) 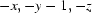. *Cg1* is the centroid of the C12*B*–C17*B* benzene ring.

**Fig. 1.**
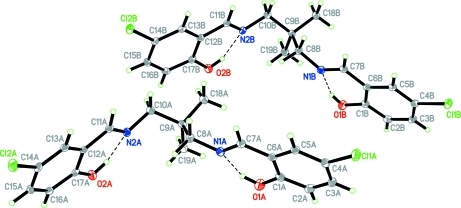
The molecular structure of (I), with atom labels and 50% probability ellipsoids for non-H atoms. Intramolecular interactions are shown as dashed lines.

**Fig. 2.**
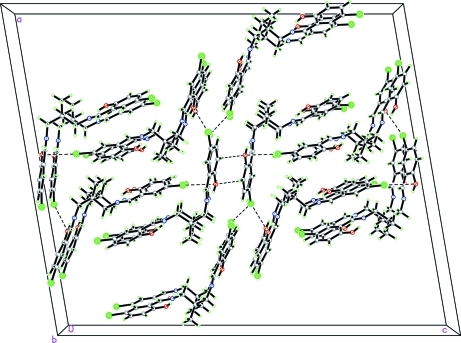
The crystal packing of (I), viewed down the b axis showing stacking of the molecules. Intermolecular interactions are shown as dashed lines.

## supplementary crystallographic information

### Comment

In the field of coordination chemistry, Schiff base is one of most prevalent versatile ligands. The Schiff base compounds have received much attention due to their important role in the development of coordination chemistry related to catalysis and enzymatic reaction, magnetism and supramolecular architectures (Casellato & Vigato 1977). In comparison to the Schiff base metal complexes, there is only a relatively small number of free Schiff base ligands which have been characterized structurally (Calligaris & Randaccio, 1987). Structures of Schiff bases derived from substituted benzaldehydes and closely related to the title compound have been reported (Li *et al.*, 2005; Bomfim *et al.*, 2005; Glidewell *et al.*, 2005, 2006; Sun *et al.*, 2004).

In the title compound (I, Fig. 1), bond lengths (Allen *et al.,* 1987) and angles are within the normal ranges and are comparable with the related bromo-substituted compound (Fun *et al.,* 2008). The asymmetric unit of (I) consists of two crystallographically independent molecules *A* and *B*. The intramolecular O—H···N hydrogen bonds generate *S*(6) ring motifs. The nitrogen atoms are also in close proximity to the hydrogen atoms of the dimethylpropane groups with H···N distances between 2.59 and 2.61 Å. The imino group is coplanar with the benzene ring. The dihedral angles between the benzene rings in molecules *A* and *B* are 58.20 (18) and 47.95 (12)°, respectively. The interesting feature of the crystal structure is the short intermolecular Cl···Cl [3.3869 (11) Å] and Cl···O [3.175 (2)– 3.204 (2) Å] interactions which are shorter than the sum of the van der Waals radii of the relevant atoms. The short distances between the centroids of the six-membered rings prove existence of π-π interactions [*Cg*1···*Cg*1^i^: 3.8711 (15) Å, (i) -*x*, -*y*, -*z*; *Cg*2···*Cg*2^ii^: 3.6424 (14) Å; (ii) 1/2 - *x*, 1/2 - *y*, - *z*; *Cg*1 and *Cg*2 are the centroids of the C12A–C17A and C12B–C17B benzene rings, respectively]. The crystal structures is further stabilized by a weak intermolecular C—H···π interaction.

### Experimental

The synthetic method has been described earlier (Fun *et al.,* 2008). Single crystals suitable for *X*-ray diffraction were obtained by evaporation of an ethanol solution at room temperature.

### Refinement

The H atoms of the hydroxy groups were located from the difference Fourier map and refined freely. The rest of the hydrogen atoms were positioned geometrically and refined using a riding model, with C—H = 0.95–0.99 Å and with *U*_iso_(H)= 1.2–1.5*U*_eq_(C). The reflection (002) was omitted as its intensity was affected by the beam backstop.

### Crystal data

**Table:**

C_19_H_20_Cl_2_N_2_O_2_	*F*(000) = 3168
*M**_r_* = 379.27	*D*_x_ = 1.368 Mg m^−^^3^
Monoclinic, *C*2/*c*	Mo *K*α radiation, λ = 0.71073 Å
Hall symbol: -C 2yc	Cell parameters from 5309 reflections
*a* = 31.6843 (8) Å	θ = 3.4–30.1°
*b* = 6.2236 (2) Å	µ = 0.37 mm^−^^1^
*c* = 37.9015 (10) Å	*T* = 100 K
β = 99.779 (1)°	Needle, yellow
*V* = 7365.2 (4) Å^3^	0.35 × 0.06 × 0.04 mm
*Z* = 16	

### Data collection

**Table:**

Bruker SMART APEXII CCD area-detector diffractometer	8427 independent reflections
Radiation source: fine-focus sealed tube	5995 reflections with *I* > 2σ(*I*)
graphite	*R*_int_ = 0.071
φ and ω scans	θ_max_ = 27.5°, θ_min_ = 1.1°
Absorption correction: multi-scan (*SADABS*; Bruker, 2005)	*h* = −40→40
*T*_min_ = 0.882, *T*_max_ = 0.986	*k* = −7→8
38685 measured reflections	*l* = −48→48

### Refinement

**Table:**

Refinement on *F*^2^	Primary atom site location: structure-invariant direct methods
Least-squares matrix: full	Secondary atom site location: difference Fourier map
*R*[*F*^2^ > 2σ(*F*^2^)] = 0.061	Hydrogen site location: inferred from neighbouring sites
*wR*(*F*^2^) = 0.116	H atoms treated by a mixture of independent and constrained refinement
*S* = 1.12	*w* = 1/[σ^2^(*F*_o_^2^) + (0.0306*P*)^2^ + 13.412*P*] where *P* = (*F*_o_^2^ + 2*F*_c_^2^)/3
8426 reflections	(Δ/σ)_max_ = 0.001
467 parameters	Δρ_max_ = 0.36 e Å^−^^3^
0 restraints	Δρ_min_ = −0.28 e Å^−^^3^

### Special details

**Table:**

Experimental. The low-temperature data was collected with the Oxford Cyrosystem Cobra low-temperature attachment.
Geometry. All esds (except the esd in the dihedral angle between two l.s. planes) are estimated using the full covariance matrix. The cell esds are taken into account individually in the estimation of esds in distances, angles and torsion angles; correlations between esds in cell parameters are only used when they are defined by crystal symmetry. An approximate (isotropic) treatment of cell esds is used for estimating esds involving l.s. planes.
Refinement. Refinement of F^2^ against ALL reflections. The weighted R-factor wR and goodness of fit S are based on F^2^, conventional R-factors R are based on F, with F set to zero for negative F^2^. The threshold expression of F^2^ > 2sigma(F^2^) is used only for calculating R-factors(gt) etc. and is not relevant to the choice of reflections for refinement. R-factors based on F^2^ are statistically about twice as large as those based on F, and R- factors based on ALL data will be even larger.

### Fractional atomic coordinates and isotropic or equivalent isotropic displacement parameters (Å^2^)

**Table:**

	*x*	*y*	*z*	*U*_iso_*/*U*_eq_	
Cl1A	0.20434 (2)	0.24028 (13)	0.342722 (18)	0.02856 (19)	
Cl2A	−0.10707 (2)	0.18921 (13)	0.037623 (19)	0.02636 (18)	
O1A	0.19084 (7)	−0.3584 (4)	0.22147 (6)	0.0279 (5)	
O2A	0.04369 (6)	−0.3662 (3)	0.04252 (5)	0.0210 (5)	
N1A	0.15011 (7)	−0.0613 (4)	0.18134 (6)	0.0181 (5)	
N2A	0.09054 (7)	−0.0464 (4)	0.06983 (5)	0.0174 (5)	
C1A	0.19459 (8)	−0.2139 (5)	0.24847 (7)	0.0192 (6)	
C2A	0.21551 (8)	−0.2762 (5)	0.28245 (7)	0.0227 (7)	
H2AA	0.2278	−0.4155	0.2859	0.027*	
C3A	0.21833 (8)	−0.1362 (5)	0.31082 (7)	0.0224 (7)	
H3AA	0.2321	−0.1802	0.3339	0.027*	
C4A	0.20119 (8)	0.0684 (5)	0.30591 (7)	0.0194 (6)	
C5A	0.18131 (8)	0.1363 (5)	0.27247 (7)	0.0183 (6)	
H5AA	0.1701	0.2780	0.2693	0.022*	
C6A	0.17774 (8)	−0.0043 (5)	0.24335 (7)	0.0169 (6)	
C7A	0.15660 (8)	0.0679 (5)	0.20798 (7)	0.0173 (6)	
H7AA	0.1475	0.2131	0.2047	0.021*	
C8A	0.12920 (8)	0.0176 (5)	0.14647 (6)	0.0180 (6)	
H8AA	0.1248	0.1746	0.1478	0.022*	
H8AB	0.1008	−0.0512	0.1400	0.022*	
C9A	0.15625 (8)	−0.0312 (5)	0.11724 (7)	0.0161 (6)	
C10A	0.13207 (8)	0.0569 (5)	0.08150 (7)	0.0205 (6)	
H10A	0.1276	0.2132	0.0840	0.025*	
H10B	0.1500	0.0365	0.0628	0.025*	
C11A	0.05717 (8)	0.0714 (5)	0.06549 (6)	0.0164 (6)	
H11A	0.0599	0.2208	0.0705	0.020*	
C12A	0.01462 (8)	−0.0191 (5)	0.05293 (6)	0.0146 (6)	
C13A	−0.02175 (8)	0.1099 (5)	0.05161 (6)	0.0166 (6)	
H13A	−0.0188	0.2542	0.0599	0.020*	
C14A	−0.06164 (8)	0.0290 (5)	0.03844 (7)	0.0185 (6)	
C15A	−0.06648 (8)	−0.1812 (5)	0.02600 (6)	0.0189 (6)	
H15A	−0.0942	−0.2355	0.0167	0.023*	
C16A	−0.03102 (8)	−0.3103 (5)	0.02717 (6)	0.0185 (6)	
H16A	−0.0343	−0.4533	0.0184	0.022*	
C17A	0.00971 (8)	−0.2331 (5)	0.04116 (6)	0.0158 (6)	
C18A	0.19908 (8)	0.0873 (5)	0.12550 (7)	0.0235 (7)	
H18A	0.2154	0.0339	0.1481	0.035*	
H18B	0.1939	0.2416	0.1276	0.035*	
H18C	0.2154	0.0622	0.1061	0.035*	
C19A	0.16397 (9)	−0.2728 (5)	0.11457 (7)	0.0236 (7)	
H19A	0.1792	−0.3260	0.1376	0.035*	
H19B	0.1813	−0.3001	0.0959	0.035*	
H19C	0.1364	−0.3470	0.1085	0.035*	
Cl1B	0.45316 (2)	0.03381 (13)	0.374929 (16)	0.02321 (17)	
Cl2B	0.16982 (2)	0.49487 (13)	0.022491 (17)	0.02330 (17)	
O1B	0.43547 (7)	−0.3355 (4)	0.23065 (5)	0.0242 (5)	
O2B	0.31272 (6)	−0.1018 (3)	0.06627 (5)	0.0214 (5)	
N1B	0.40114 (7)	0.0212 (4)	0.20579 (5)	0.0183 (5)	
N2B	0.35608 (7)	0.2186 (4)	0.09782 (5)	0.0179 (5)	
C1B	0.43973 (8)	−0.2439 (5)	0.26335 (7)	0.0174 (6)	
C2B	0.45942 (8)	−0.3620 (5)	0.29294 (7)	0.0198 (6)	
H2BA	0.4700	−0.5021	0.2896	0.024*	
C3B	0.46363 (8)	−0.2767 (5)	0.32686 (7)	0.0190 (6)	
H3BA	0.4771	−0.3576	0.3469	0.023*	
C4B	0.44814 (8)	−0.0720 (5)	0.33167 (6)	0.0174 (6)	
C5B	0.42920 (8)	0.0493 (5)	0.30294 (6)	0.0165 (6)	
H5BA	0.4190	0.1897	0.3067	0.020*	
C6B	0.42492 (8)	−0.0344 (5)	0.26820 (7)	0.0160 (6)	
C7B	0.40641 (8)	0.0972 (5)	0.23753 (7)	0.0166 (6)	
H7BA	0.3982	0.2411	0.2412	0.020*	
C8B	0.38462 (8)	0.1593 (5)	0.17565 (6)	0.0181 (6)	
H8BA	0.3800	0.3056	0.1845	0.022*	
H8BB	0.3567	0.1032	0.1635	0.022*	
C9B	0.41600 (8)	0.1705 (5)	0.14872 (6)	0.0155 (6)	
C10B	0.39544 (8)	0.3118 (5)	0.11726 (7)	0.0182 (6)	
H10C	0.3891	0.4550	0.1264	0.022*	
H10D	0.4161	0.3318	0.1006	0.022*	
C11B	0.32341 (8)	0.3398 (5)	0.08917 (6)	0.0168 (6)	
H11B	0.3248	0.4860	0.0965	0.020*	
C12B	0.28383 (8)	0.2557 (5)	0.06813 (6)	0.0154 (6)	
C13B	0.24910 (8)	0.3932 (5)	0.05820 (6)	0.0166 (6)	
H13B	0.2504	0.5372	0.0666	0.020*	
C14B	0.21274 (8)	0.3199 (5)	0.03607 (6)	0.0162 (6)	
C15B	0.21020 (8)	0.1105 (5)	0.02371 (6)	0.0179 (6)	
H15B	0.1854	0.0631	0.0079	0.021*	
C16B	0.24363 (8)	−0.0291 (5)	0.03426 (6)	0.0183 (6)	
H16B	0.2414	−0.1739	0.0262	0.022*	
C17B	0.28073 (8)	0.0403 (5)	0.05664 (6)	0.0167 (6)	
C18B	0.45789 (8)	0.2777 (5)	0.16634 (7)	0.0199 (6)	
H18D	0.4517	0.4214	0.1747	0.030*	
H18E	0.4773	0.2895	0.1488	0.030*	
H18F	0.4714	0.1906	0.1867	0.030*	
C19B	0.42487 (9)	−0.0548 (5)	0.13544 (7)	0.0211 (6)	
H19D	0.3979	−0.1213	0.1243	0.032*	
H19E	0.4383	−0.1428	0.1557	0.032*	
H19F	0.4441	−0.0443	0.1178	0.032*	
H1OA	0.1757 (12)	−0.292 (7)	0.2017 (10)	0.059 (12)*	
H2OA	0.0665 (11)	−0.295 (6)	0.0505 (9)	0.046 (11)*	
H1OB	0.4234 (11)	−0.242 (6)	0.2164 (9)	0.041 (11)*	
H2OB	0.3331 (11)	−0.033 (6)	0.0771 (9)	0.048 (12)*	

### Atomic displacement parameters (Å^2^)

**Table:**

	*U*^11^	*U*^22^	*U*^33^	*U*^12^	*U*^13^	*U*^23^
Cl1A	0.0378 (4)	0.0266 (4)	0.0191 (3)	0.0004 (4)	−0.0016 (3)	−0.0006 (3)
Cl2A	0.0180 (3)	0.0278 (4)	0.0317 (4)	0.0071 (3)	−0.0004 (3)	0.0009 (4)
O1A	0.0364 (12)	0.0192 (12)	0.0271 (11)	0.0081 (11)	0.0025 (9)	−0.0016 (10)
O2A	0.0201 (10)	0.0174 (12)	0.0245 (10)	0.0017 (10)	0.0011 (8)	−0.0052 (9)
N1A	0.0165 (11)	0.0191 (14)	0.0188 (11)	−0.0005 (11)	0.0029 (9)	−0.0013 (11)
N2A	0.0174 (11)	0.0217 (14)	0.0127 (10)	−0.0015 (11)	0.0009 (8)	−0.0007 (10)
C1A	0.0142 (12)	0.0186 (16)	0.0252 (13)	−0.0005 (13)	0.0045 (10)	−0.0012 (13)
C2A	0.0185 (13)	0.0193 (17)	0.0300 (15)	0.0043 (14)	0.0035 (11)	0.0063 (14)
C3A	0.0144 (13)	0.0275 (18)	0.0237 (14)	0.0009 (14)	−0.0012 (11)	0.0089 (14)
C4A	0.0145 (12)	0.0216 (17)	0.0214 (13)	−0.0013 (13)	0.0008 (10)	0.0007 (13)
C5A	0.0180 (13)	0.0146 (15)	0.0220 (13)	0.0005 (13)	0.0029 (10)	0.0031 (13)
C6A	0.0121 (11)	0.0176 (16)	0.0211 (13)	−0.0019 (13)	0.0035 (10)	0.0030 (13)
C7A	0.0140 (12)	0.0183 (16)	0.0203 (13)	0.0016 (13)	0.0056 (10)	0.0021 (13)
C8A	0.0153 (12)	0.0192 (16)	0.0193 (12)	0.0027 (13)	0.0024 (10)	−0.0013 (13)
C9A	0.0141 (12)	0.0174 (16)	0.0175 (12)	0.0002 (13)	0.0046 (10)	−0.0020 (12)
C10A	0.0156 (13)	0.0249 (18)	0.0214 (13)	−0.0032 (13)	0.0039 (10)	0.0011 (13)
C11A	0.0218 (13)	0.0151 (16)	0.0129 (12)	−0.0022 (13)	0.0045 (10)	0.0000 (12)
C12A	0.0204 (13)	0.0134 (15)	0.0097 (11)	−0.0026 (13)	0.0011 (9)	0.0015 (11)
C13A	0.0208 (13)	0.0128 (15)	0.0158 (12)	0.0005 (13)	0.0022 (10)	0.0007 (12)
C14A	0.0186 (13)	0.0210 (17)	0.0158 (12)	0.0072 (14)	0.0023 (10)	0.0048 (13)
C15A	0.0177 (13)	0.0241 (17)	0.0134 (12)	−0.0055 (14)	−0.0012 (10)	−0.0001 (12)
C16A	0.0264 (14)	0.0143 (15)	0.0143 (12)	−0.0021 (14)	0.0024 (10)	0.0005 (12)
C17A	0.0196 (13)	0.0176 (16)	0.0104 (11)	0.0017 (13)	0.0031 (9)	0.0027 (12)
C18A	0.0191 (13)	0.0257 (18)	0.0252 (14)	−0.0034 (14)	0.0022 (11)	0.0003 (14)
C19A	0.0221 (14)	0.0248 (18)	0.0241 (14)	0.0006 (15)	0.0043 (11)	−0.0056 (14)
Cl1B	0.0253 (3)	0.0287 (4)	0.0162 (3)	0.0025 (4)	0.0053 (2)	0.0007 (3)
Cl2B	0.0172 (3)	0.0252 (4)	0.0255 (3)	0.0033 (3)	−0.0020 (2)	0.0031 (3)
O1B	0.0327 (12)	0.0183 (12)	0.0210 (10)	0.0053 (11)	0.0030 (9)	−0.0026 (10)
O2B	0.0213 (10)	0.0169 (12)	0.0247 (10)	0.0035 (10)	−0.0001 (8)	−0.0025 (9)
N1B	0.0176 (11)	0.0182 (14)	0.0192 (11)	−0.0006 (11)	0.0033 (9)	0.0011 (11)
N2B	0.0188 (11)	0.0189 (14)	0.0150 (10)	−0.0013 (11)	0.0001 (8)	0.0029 (10)
C1B	0.0132 (12)	0.0186 (16)	0.0210 (13)	0.0005 (13)	0.0043 (10)	−0.0004 (13)
C2B	0.0161 (13)	0.0148 (16)	0.0291 (14)	0.0034 (13)	0.0057 (11)	0.0010 (13)
C3B	0.0134 (12)	0.0211 (17)	0.0226 (13)	0.0030 (13)	0.0029 (10)	0.0079 (13)
C4B	0.0153 (12)	0.0223 (17)	0.0158 (12)	−0.0022 (13)	0.0065 (10)	0.0001 (12)
C5B	0.0144 (12)	0.0156 (15)	0.0207 (12)	0.0008 (12)	0.0061 (10)	0.0001 (12)
C6B	0.0127 (12)	0.0157 (15)	0.0202 (12)	−0.0008 (13)	0.0043 (10)	0.0018 (12)
C7B	0.0129 (12)	0.0147 (15)	0.0231 (13)	0.0001 (12)	0.0052 (10)	0.0006 (12)
C8B	0.0141 (12)	0.0217 (17)	0.0181 (12)	0.0024 (13)	0.0017 (10)	0.0035 (13)
C9B	0.0134 (12)	0.0156 (15)	0.0170 (12)	0.0005 (12)	0.0014 (10)	−0.0013 (12)
C10B	0.0174 (13)	0.0181 (16)	0.0186 (12)	−0.0015 (13)	0.0019 (10)	0.0010 (12)
C11B	0.0224 (14)	0.0159 (15)	0.0123 (11)	−0.0023 (13)	0.0034 (10)	0.0009 (12)
C12B	0.0182 (13)	0.0171 (15)	0.0117 (11)	−0.0002 (13)	0.0047 (10)	0.0011 (12)
C13B	0.0193 (13)	0.0150 (15)	0.0162 (12)	0.0008 (13)	0.0049 (10)	0.0025 (12)
C14B	0.0163 (12)	0.0187 (16)	0.0142 (12)	0.0037 (13)	0.0039 (10)	0.0049 (12)
C15B	0.0170 (13)	0.0253 (17)	0.0116 (12)	−0.0068 (13)	0.0034 (10)	−0.0016 (12)
C16B	0.0239 (14)	0.0157 (16)	0.0165 (12)	−0.0027 (14)	0.0065 (10)	−0.0013 (12)
C17B	0.0204 (13)	0.0185 (16)	0.0125 (11)	0.0021 (13)	0.0065 (10)	0.0011 (12)
C18B	0.0159 (13)	0.0211 (17)	0.0220 (13)	−0.0005 (13)	0.0012 (10)	−0.0019 (13)
C19B	0.0224 (14)	0.0194 (17)	0.0205 (13)	0.0013 (14)	0.0012 (11)	−0.0017 (13)

### Geometric parameters (Å, °)

**Table:**

Cl1A—C4A	1.747 (3)	Cl1B—C4B	1.749 (3)
Cl2A—C14A	1.747 (3)	Cl2B—C14B	1.750 (3)
O1A—C1A	1.352 (3)	O1B—C1B	1.350 (3)
O1A—H1OA	0.92 (4)	O1B—H1OB	0.84 (4)
O2A—C17A	1.352 (3)	O2B—C17B	1.348 (3)
O2A—H2OA	0.86 (4)	O2B—H2OB	0.82 (4)
N1A—C7A	1.280 (3)	N1B—C7B	1.277 (3)
N1A—C8A	1.459 (3)	N1B—C8B	1.454 (3)
N2A—C11A	1.274 (3)	N2B—C11B	1.278 (3)
N2A—C10A	1.464 (3)	N2B—C10B	1.457 (3)
C1A—C2A	1.400 (4)	C1B—C2B	1.396 (4)
C1A—C6A	1.410 (4)	C1B—C6B	1.408 (4)
C2A—C3A	1.375 (4)	C2B—C3B	1.376 (4)
C2A—H2AA	0.9500	C2B—H2BA	0.9500
C3A—C4A	1.385 (4)	C3B—C4B	1.388 (4)
C3A—H3AA	0.9500	C3B—H3BA	0.9500
C4A—C5A	1.382 (3)	C4B—C5B	1.376 (4)
C5A—C6A	1.398 (4)	C5B—C6B	1.401 (3)
C5A—H5AA	0.9500	C5B—H5BA	0.9500
C6A—C7A	1.464 (3)	C6B—C7B	1.461 (4)
C7A—H7AA	0.9500	C7B—H7BA	0.9500
C8A—C9A	1.542 (3)	C8B—C9B	1.543 (3)
C8A—H8AA	0.9900	C8B—H8BA	0.9900
C8A—H8AB	0.9900	C8B—H8BB	0.9900
C9A—C18A	1.529 (4)	C9B—C19B	1.531 (4)
C9A—C19A	1.529 (4)	C9B—C18B	1.534 (3)
C9A—C10A	1.539 (3)	C9B—C10B	1.535 (4)
C10A—H10A	0.9900	C10B—H10C	0.9900
C10A—H10B	0.9900	C10B—H10D	0.9900
C11A—C12A	1.464 (3)	C11B—C12B	1.464 (3)
C11A—H11A	0.9500	C11B—H11B	0.9500
C12A—C13A	1.398 (4)	C12B—C13B	1.394 (4)
C12A—C17A	1.405 (4)	C12B—C17B	1.408 (4)
C13A—C14A	1.373 (4)	C13B—C14B	1.382 (3)
C13A—H13A	0.9500	C13B—H13B	0.9500
C14A—C15A	1.390 (4)	C14B—C15B	1.383 (4)
C15A—C16A	1.376 (4)	C15B—C16B	1.376 (4)
C15A—H15A	0.9500	C15B—H15B	0.9500
C16A—C17A	1.395 (4)	C16B—C17B	1.396 (4)
C16A—H16A	0.9500	C16B—H16B	0.9500
C18A—H18A	0.9800	C18B—H18D	0.9800
C18A—H18B	0.9800	C18B—H18E	0.9800
C18A—H18C	0.9800	C18B—H18F	0.9800
C19A—H19A	0.9800	C19B—H19D	0.9800
C19A—H19B	0.9800	C19B—H19E	0.9800
C19A—H19C	0.9800	C19B—H19F	0.9800
			
C1A—O1A—H1OA	107 (2)	C1B—O1B—H1OB	105 (2)
C17A—O2A—H2OA	108 (2)	C17B—O2B—H2OB	107 (3)
C7A—N1A—C8A	119.4 (2)	C7B—N1B—C8B	119.6 (3)
C11A—N2A—C10A	118.0 (2)	C11B—N2B—C10B	118.8 (2)
O1A—C1A—C2A	118.6 (3)	O1B—C1B—C2B	118.5 (3)
O1A—C1A—C6A	121.9 (2)	O1B—C1B—C6B	121.8 (2)
C2A—C1A—C6A	119.5 (3)	C2B—C1B—C6B	119.7 (2)
C3A—C2A—C1A	120.1 (3)	C3B—C2B—C1B	120.4 (3)
C3A—C2A—H2AA	119.9	C3B—C2B—H2BA	119.8
C1A—C2A—H2AA	119.9	C1B—C2B—H2BA	119.8
C2A—C3A—C4A	120.4 (2)	C2B—C3B—C4B	119.8 (3)
C2A—C3A—H3AA	119.8	C2B—C3B—H3BA	120.1
C4A—C3A—H3AA	119.8	C4B—C3B—H3BA	120.1
C5A—C4A—C3A	120.8 (3)	C5B—C4B—C3B	121.1 (2)
C5A—C4A—Cl1A	120.0 (2)	C5B—C4B—Cl1B	119.6 (2)
C3A—C4A—Cl1A	119.2 (2)	C3B—C4B—Cl1B	119.4 (2)
C4A—C5A—C6A	119.7 (3)	C4B—C5B—C6B	120.0 (3)
C4A—C5A—H5AA	120.1	C4B—C5B—H5BA	120.0
C6A—C5A—H5AA	120.1	C6B—C5B—H5BA	120.0
C5A—C6A—C1A	119.5 (2)	C5B—C6B—C1B	119.1 (2)
C5A—C6A—C7A	119.6 (3)	C5B—C6B—C7B	120.1 (3)
C1A—C6A—C7A	120.9 (3)	C1B—C6B—C7B	120.8 (2)
N1A—C7A—C6A	121.1 (3)	N1B—C7B—C6B	120.8 (3)
N1A—C7A—H7AA	119.4	N1B—C7B—H7BA	119.6
C6A—C7A—H7AA	119.4	C6B—C7B—H7BA	119.6
N1A—C8A—C9A	111.3 (2)	N1B—C8B—C9B	111.1 (2)
N1A—C8A—H8AA	109.4	N1B—C8B—H8BA	109.4
C9A—C8A—H8AA	109.4	C9B—C8B—H8BA	109.4
N1A—C8A—H8AB	109.4	N1B—C8B—H8BB	109.4
C9A—C8A—H8AB	109.4	C9B—C8B—H8BB	109.4
H8AA—C8A—H8AB	108.0	H8BA—C8B—H8BB	108.0
C18A—C9A—C19A	110.0 (2)	C19B—C9B—C18B	110.4 (2)
C18A—C9A—C10A	107.5 (2)	C19B—C9B—C10B	110.3 (2)
C19A—C9A—C10A	110.7 (2)	C18B—C9B—C10B	108.1 (2)
C18A—C9A—C8A	109.9 (2)	C19B—C9B—C8B	110.6 (2)
C19A—C9A—C8A	110.8 (2)	C18B—C9B—C8B	109.8 (2)
C10A—C9A—C8A	107.9 (2)	C10B—C9B—C8B	107.7 (2)
N2A—C10A—C9A	113.4 (2)	N2B—C10B—C9B	112.2 (2)
N2A—C10A—H10A	108.9	N2B—C10B—H10C	109.2
C9A—C10A—H10A	108.9	C9B—C10B—H10C	109.2
N2A—C10A—H10B	108.9	N2B—C10B—H10D	109.2
C9A—C10A—H10B	108.9	C9B—C10B—H10D	109.2
H10A—C10A—H10B	107.7	H10C—C10B—H10D	107.9
N2A—C11A—C12A	121.2 (3)	N2B—C11B—C12B	120.7 (3)
N2A—C11A—H11A	119.4	N2B—C11B—H11B	119.7
C12A—C11A—H11A	119.4	C12B—C11B—H11B	119.7
C13A—C12A—C17A	119.1 (2)	C13B—C12B—C17B	119.4 (2)
C13A—C12A—C11A	119.9 (3)	C13B—C12B—C11B	119.4 (3)
C17A—C12A—C11A	121.0 (2)	C17B—C12B—C11B	121.2 (2)
C14A—C13A—C12A	120.3 (3)	C14B—C13B—C12B	120.0 (3)
C14A—C13A—H13A	119.8	C14B—C13B—H13B	120.0
C12A—C13A—H13A	119.8	C12B—C13B—H13B	120.0
C13A—C14A—C15A	120.6 (3)	C13B—C14B—C15B	120.7 (3)
C13A—C14A—Cl2A	120.1 (2)	C13B—C14B—Cl2B	119.9 (2)
C15A—C14A—Cl2A	119.2 (2)	C15B—C14B—Cl2B	119.3 (2)
C16A—C15A—C14A	119.8 (2)	C16B—C15B—C14B	120.0 (2)
C16A—C15A—H15A	120.1	C16B—C15B—H15B	120.0
C14A—C15A—H15A	120.1	C14B—C15B—H15B	120.0
C15A—C16A—C17A	120.6 (3)	C15B—C16B—C17B	120.6 (3)
C15A—C16A—H16A	119.7	C15B—C16B—H16B	119.7
C17A—C16A—H16A	119.7	C17B—C16B—H16B	119.7
O2A—C17A—C16A	118.9 (3)	O2B—C17B—C16B	118.6 (3)
O2A—C17A—C12A	121.6 (2)	O2B—C17B—C12B	122.1 (2)
C16A—C17A—C12A	119.5 (3)	C16B—C17B—C12B	119.3 (3)
C9A—C18A—H18A	109.5	C9B—C18B—H18D	109.5
C9A—C18A—H18B	109.5	C9B—C18B—H18E	109.5
H18A—C18A—H18B	109.5	H18D—C18B—H18E	109.5
C9A—C18A—H18C	109.5	C9B—C18B—H18F	109.5
H18A—C18A—H18C	109.5	H18D—C18B—H18F	109.5
H18B—C18A—H18C	109.5	H18E—C18B—H18F	109.5
C9A—C19A—H19A	109.5	C9B—C19B—H19D	109.5
C9A—C19A—H19B	109.5	C9B—C19B—H19E	109.5
H19A—C19A—H19B	109.5	H19D—C19B—H19E	109.5
C9A—C19A—H19C	109.5	C9B—C19B—H19F	109.5
H19A—C19A—H19C	109.5	H19D—C19B—H19F	109.5
H19B—C19A—H19C	109.5	H19E—C19B—H19F	109.5
			
O1A—C1A—C2A—C3A	−177.3 (3)	O1B—C1B—C2B—C3B	−178.6 (2)
C6A—C1A—C2A—C3A	2.1 (4)	C6B—C1B—C2B—C3B	1.4 (4)
C1A—C2A—C3A—C4A	−1.1 (4)	C1B—C2B—C3B—C4B	0.1 (4)
C2A—C3A—C4A—C5A	−0.6 (4)	C2B—C3B—C4B—C5B	−1.1 (4)
C2A—C3A—C4A—Cl1A	178.7 (2)	C2B—C3B—C4B—Cl1B	179.4 (2)
C3A—C4A—C5A—C6A	1.2 (4)	C3B—C4B—C5B—C6B	0.7 (4)
Cl1A—C4A—C5A—C6A	−178.0 (2)	Cl1B—C4B—C5B—C6B	−179.81 (19)
C4A—C5A—C6A—C1A	−0.2 (4)	C4B—C5B—C6B—C1B	0.8 (4)
C4A—C5A—C6A—C7A	179.9 (2)	C4B—C5B—C6B—C7B	−177.5 (2)
O1A—C1A—C6A—C5A	177.9 (2)	O1B—C1B—C6B—C5B	178.2 (2)
C2A—C1A—C6A—C5A	−1.5 (4)	C2B—C1B—C6B—C5B	−1.8 (4)
O1A—C1A—C6A—C7A	−2.1 (4)	O1B—C1B—C6B—C7B	−3.5 (4)
C2A—C1A—C6A—C7A	178.5 (2)	C2B—C1B—C6B—C7B	176.5 (2)
C8A—N1A—C7A—C6A	−179.6 (2)	C8B—N1B—C7B—C6B	−176.9 (2)
C5A—C6A—C7A—N1A	−175.0 (2)	C5B—C6B—C7B—N1B	−177.9 (2)
C1A—C6A—C7A—N1A	5.0 (4)	C1B—C6B—C7B—N1B	3.8 (4)
C7A—N1A—C8A—C9A	126.1 (3)	C7B—N1B—C8B—C9B	122.3 (3)
N1A—C8A—C9A—C18A	−63.0 (3)	N1B—C8B—C9B—C19B	57.4 (3)
N1A—C8A—C9A—C19A	58.7 (3)	N1B—C8B—C9B—C18B	−64.6 (3)
N1A—C8A—C9A—C10A	−180.0 (2)	N1B—C8B—C9B—C10B	177.9 (2)
C11A—N2A—C10A—C9A	120.4 (3)	C11B—N2B—C10B—C9B	135.0 (2)
C18A—C9A—C10A—N2A	178.7 (2)	C19B—C9B—C10B—N2B	57.0 (3)
C19A—C9A—C10A—N2A	58.7 (3)	C18B—C9B—C10B—N2B	177.7 (2)
C8A—C9A—C10A—N2A	−62.8 (3)	C8B—C9B—C10B—N2B	−63.7 (3)
C10A—N2A—C11A—C12A	178.3 (2)	C10B—N2B—C11B—C12B	177.0 (2)
N2A—C11A—C12A—C13A	174.1 (2)	N2B—C11B—C12B—C13B	−178.2 (2)
N2A—C11A—C12A—C17A	−7.7 (4)	N2B—C11B—C12B—C17B	−0.5 (4)
C17A—C12A—C13A—C14A	−1.0 (4)	C17B—C12B—C13B—C14B	−2.6 (4)
C11A—C12A—C13A—C14A	177.2 (2)	C11B—C12B—C13B—C14B	175.1 (2)
C12A—C13A—C14A—C15A	−0.5 (4)	C12B—C13B—C14B—C15B	0.3 (4)
C12A—C13A—C14A—Cl2A	178.79 (19)	C12B—C13B—C14B—Cl2B	−178.03 (19)
C13A—C14A—C15A—C16A	0.6 (4)	C13B—C14B—C15B—C16B	1.9 (4)
Cl2A—C14A—C15A—C16A	−178.70 (19)	Cl2B—C14B—C15B—C16B	−179.76 (19)
C14A—C15A—C16A—C17A	0.8 (4)	C14B—C15B—C16B—C17B	−1.7 (4)
C15A—C16A—C17A—O2A	178.7 (2)	C15B—C16B—C17B—O2B	−179.9 (2)
C15A—C16A—C17A—C12A	−2.3 (4)	C15B—C16B—C17B—C12B	−0.6 (4)
C13A—C12A—C17A—O2A	−178.6 (2)	C13B—C12B—C17B—O2B	−178.0 (2)
C11A—C12A—C17A—O2A	3.1 (4)	C11B—C12B—C17B—O2B	4.4 (4)
C13A—C12A—C17A—C16A	2.4 (4)	C13B—C12B—C17B—C16B	2.7 (4)
C11A—C12A—C17A—C16A	−175.8 (2)	C11B—C12B—C17B—C16B	−174.9 (2)

### Hydrogen-bond geometry (Å, °)

**Table:**

*D*—H···*A*	*D*—H	H···*A*	*D*···*A*	*D*—H···*A*
O1A—H1OA···N1A	0.92 (4)	1.76 (4)	2.594 (3)	150 (4)
O2A—H2OA···N2A	0.86 (4)	1.82 (4)	2.591 (3)	148 (3)
O1B—H1OB···N1B	0.84 (4)	1.80 (4)	2.579 (3)	153 (3)
O2B—H2OB···N2B	0.82 (4)	1.85 (4)	2.595 (3)	151 (4)
C16A—H16A···O2A^i^	0.95	2.54	3.291 (3)	136
C18A—H18C···Cg1	0.98	2.73	3.634 (3)	153

Symmetry codes: (i) −*x*, −*y*−1, −*z*.
